# Frailty Alerts Reduce Waiting Time and Length of Stay in the Emergency Department

**DOI:** 10.1111/acem.70239

**Published:** 2026-02-06

**Authors:** Samia Munir Ehrlington, Jens Wretborn, Daniel Wilhelms

**Affiliations:** ^1^ Clinical Department of Emergency Medicine Linköping University Linköping Sweden; ^2^ Department of Biomedical and Clinical Sciences Linköping University Linköping Sweden

## Abstract

**Background:**

Prolonged emergency department waiting times are associated with increased mortality among older patients. In January 2025, the ED of Linkoping University Hospital, Sweden, implemented a low‐resource routine to expedite the workup of older patients living with frailty by prioritized physician assessment and subsequent workup.

**Aim:**

To investigate if a frailty alert using the Clinical Frailty Scale followed by prioritized clinical assessment influences ED operating metrics.

**Design:**

This was an observational before and after study of a pre‐implementation group (control) and a post‐implementation group (intervention) between October 2024 and February 2025.

**Setting/Participants:**

Consecutive patients aged > 64 years, with a documented CFS assessment during the ED visit at the Linkoping University Hospital, Sweden, who consented to participation, were included.

**Method:**

Standard ED operating metrics, *Time to physician*, *ED length of stay* (LOS), and *admission rates* were compared between a pre‐implementation group and a post‐implementation group.

**Results:**

A total of 542 ED visits were analyzed (248 pre‐implementation, 294 post‐implementation). *Time to physician* was shorter in the post‐implementation group at 31 min (IQR 15, 65) versus 44 min (IQR 20, 94) (*p* < 0.001). *ED LOS* was reduced from 352 (IQR 266, 515) to 319 (IQR 240, 458) minutes (*p* = 0.014). The *admission rate* was unchanged at 59% and 60% (*p* = 0.4).

**Conclusion:**

Frailty alerts based on the CFS with prioritized workup reduced *ED LOS* and *time to physician* in older patients living with frailty in this single center study and may be a low‐resource intervention to reduce the risks of adverse events in the ED.

**Trial Registration:**

ClinicalTrials.gov identifier: NCT06869148

## 1 Background

Emergency departments (ED) face challenges from an increasingly aging population with an expected fivefold increase of ED presentations among those aged ≥ 86 years between 2010 and 2050 [1, 2, 3]. In Sweden, patients aged ≥ 65 years constitute around 20% of the population whilst accounting for 40% of all ED visits [4]. Older patients often wait longer for physician initial assessment [5, 6] more commonly display complex clinical presentations, and generally utilize more ED resources [7, 8], partly due to atypical presentations and multiple comorbidities [7] requiring extensive workup [9, 10, 11]. Atypical presentations with nonspecific complaints recur frequently in older patients living with frailty [12], a syndrome characterized by decreased physiological reserve capacity [13]. Frailty has consistently been shown to predict adverse outcomes in the ED across diverse healthcare settings. Older adults living with frailty experience prolonged ED length of stay (ED LOS) [14, 15, 16], higher admission rates and longer hospital stay [13, 14, 16, 17], functional decline [12, 13, 17] and short‐term mortality [14, 16, 17]. There is an association between extended ED LOS and higher rates of in‐hospital mortality in patients with limited autonomy and who are living with frailty [16, 18, 19, 20]. Prolonged ED LOS among older adults may be precipitated by the exclusive reliance on standard triage systems for initial risk assessment, as these tools have a well‐documented bias toward undertriage in this patient population [21, 22, 23], thereby delaying both initial physician evaluation and subsequent diagnostic workup. This creates a downstream of delays that compounds in time, causing unnecessary delays and ultimately increasing the risk of adverse effects.

Among ED interventions aimed to reduce adverse events in older patients, specialized geriatric teams in the ED have been shown to reduce avoidable hospital admissions, ED re‐attendance, and functional decline [24, 25]. Most interventions, however, fail to improve operational or patient related outcomes [26, 27]. Despite recommendations for early frailty assessment in emergency care settings [28], implementation and effectiveness of frailty alert systems remain insufficiently investigated. While whiteboard flagging using the Clinical Frailty Scale has facilitated staff prioritization of care [29], and electronic Clinical Frailty Scale alerts have enhanced timeliness of Comprehensive Geriatric Assessment for direct admissions to frailty assessment units [30], the impact of frailty alerts in a general ED without specialized geriatric resources remains a gap in our current knowledge.

At our general ED, at the Linköping University Hospital in Sweden, frailty assessments for elderly patients have been a clinical routine since 2021 and we have shown that frailty is associated with mortality, hospital admission and increased length of stay [14]. As a result, an addition to the clinical routine was introduced in January 2025 to act on the frailty assessment. The routine recommends that all patients aged > 64 years of age were recommended to receive an early CFS assessment and, if the patient was assessed as living with frailty (CFS > 4), a frailty alert would be documented in the electronic ledger where all current patients in the ED are displayed for the clinicians. Patients with frailty alert were to be prioritized for assessment by physicians and a plan for care to prevent possible iatrogenic complications such as delirium and falls was to be established according to guideline recommendations and patients' current status.

This study investigated whether the intervention of a frailty alert system with prioritized clinical workup affected emergency department length of stay and time to physician assessment in older patients living with frailty. The study also examined potential displacement effects on robust older patients attending the emergency department during the same period.

## 2 Method

### 2.1 Study Design and Setting

This was an observational, retrospective, before‐after study comparing outcomes between a pre‐implementation control period and a post‐implementation intervention period. The study was carried out in the ED at Linköping University hospital, an urban tertiary care center serving a population of approximately 170,000 inhabitants in Sweden. The ED receives around 50,000 visits annually. Patients aged > 64 years of age contribute to around 30% of these visits and approximately 50% of all hospital admissions from the ED. The intervention comprised early frailty identification using the Clinical Frailty Scale (CFS), preferably during triage, with documentation of frailty alerts (CFS > 4) in the electronic ED ledger, followed by prioritized physician assessment and targeted care planning by the treating team consisting of an emergency physician, a registered nurse, and an assistant nurse. Clinical outcomes were compared between a pre‐implementation group (control period) and post‐implementation group (intervention period). The data collection period occurred over 6 weeks in October–December, 2024 (pre‐implementation group) and over 6 weeks in January–March, 2025 (post‐implementation group). The study protocol was prospectively registered on ClinicalTrials.gov and the study was approved by the Swedish Ethical Review Authority (reference no: 2024‐05740‐01). No other relevant changes in ED operations were made during the study period.

### 2.2 Selection of Participants

All patients visiting the ED during the study period aged > 64 years with a CFS score documented in the electronic health records were eligible for inclusion. Written information about the study and the possibility to opt out was sent by mail to all eligible patients. The patient or a proxy (a caretaker/next of kin) could opt out either by mail, email, or by telephone. In addition, patients were excluded who did not receive the opt out information (written information returned to the research team) and were not deceased according to the Swedish population registry.

### 2.3 Outcome Measures

The primary outcome was ED length of stay. Secondary outcomes were: time to the first assessment by a physician, admission rate, and difference in ED length of stay between patients in different triage categories. All outcomes were compared between the pre‐implementation and post‐implementation period for the robust patients as well to investigate potential displacement effects.

### 2.4 Statistical Analysis

#### 2.4.1 Data Analysis and Sample Size Calculation

Sample size calculation aimed to reduce length of stay for patients living with frailty in triage category 3 (urgent) to match that of robust patients aged > 64 years in triage category 2 (very urgent). Previous data collection in the same setting showed that triage category 3 contained the largest proportion of older patients living with frailty [14]. In the previous study cohort, 50% of patients > 64 years of age were assessed as CFS > 4 and the difference in ED LOS between patients with frailty in triage category 3 compared to robust patients in triage category 2 was 60 min. Given that the ED LOS varies less in the highest and lowest triage categories, we estimated that a realistic target in effect size would be a 45‐min reduction in LOS for patients living with frailty.

With an *α* level of 0.05, power of 0.8, an effect size of 45 min with a standard deviation of 150 min, a total of 176 patients were needed for follow‐up in the post‐implementation group. To account for approximately 20% loss to follow up and sub‐group analysis, we aimed to include 240 patients with CFS > 4 in the pre‐ and post‐implementation groups respectively. To explore possible secondary displacement effects of the clinical routine on robust ED patients (CFS < 5), the outcome measures were investigated for this group as well. With patients aged > 64 living with frailty constituting one third of assessed patients with 50% being CFS > 4, we aimed for 960–1440 for the total study population [14]. Data collection was finalized when the targeted number of patients with CFS > 4 was reached and no opting‐out had been made within 5 weeks of receiving the study information, as stated in the ethics approval.

Descriptive statistics were reported as medians for continuous variables and percentages for categorical variables. Median for LOS was compared using the Wilcoxon rank sum test, and the admission rate was compared using Pearson's Chi‐squared test or Fisher's exact test when the number of outcomes was lower than five in any group.

#### 2.4.2 Patient and Public Involvement

Research partners aged ≥ 65 years from a local patient involvement organization contributed to study design review and outcome selection. Further into the planning process, the research partners participated in the development of patient information materials, provided advice, and contributed information material and method of delivery.

## 3 Results

### 3.1 Characteristics of the Study Subjects

**FIGURE 1:**
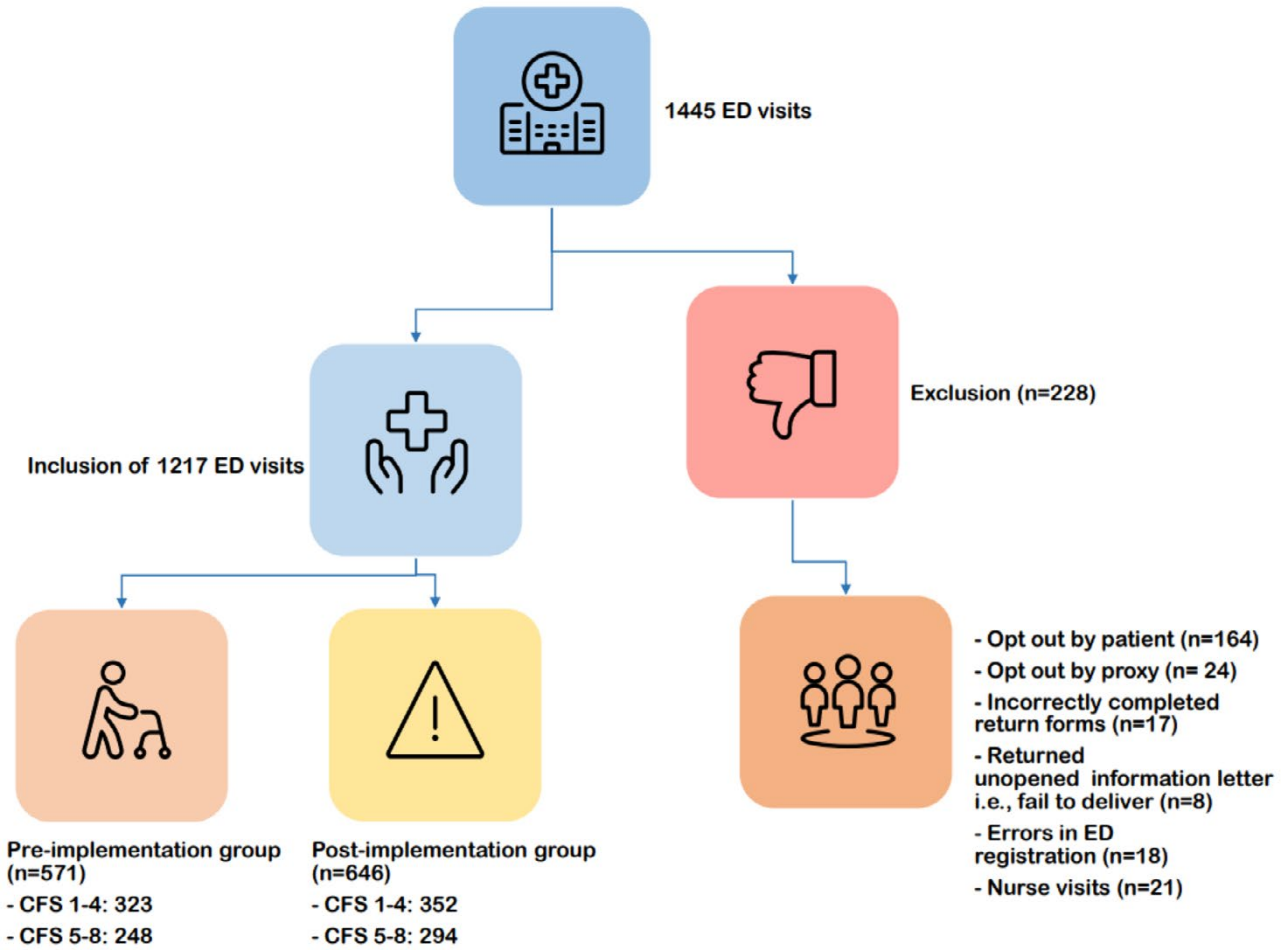
Flowchart describing the inclusion process. CFS, Clinical Frailty Scale; ED, emergency department.

After exclusion of 228 patients, a total of 542 ED visits of patients living with frailty with > 64 years of age were included in the study, with 248 visits in the pre‐implementation group and 294 visits in the post‐implementation group (Figure 1). The proportion of patients assessed as CFS > 4 was 43.2% in the pre‐implementation group and 45% in the post‐implementation group. The mean age was similar for patients living with frailty in both cohorts (85 and 84 years) and the proportion of women was similar at 57% in the pre‐implementation group and 55% in the post‐implementation group (Table 1). In the robust groups, women constituted 47% in the pre‐implementation group and 50% in the post‐implementation group.

**TABLE 1:** Basic characteristics of the study cohort.

	Pre‐implementation group^a^	Post‐implementation group^a^
	Living with frailty (CFS > 4)	Living with frailty (CFS > 4)
*n*	248	294
Age, median (IQR)	84 (79, 89)	85 (79, 90)
Women (%)	142 (57%)	163 (55%)
CFS		
5	114 (46%)	142 (48%)
6	78 (31%)	78 (27%)
7	54 (22%)	72 (25%)
8	2 (0.8%)	2 (0.7%)
Mode of arrival		
Ambulance	162 (65%)	203 (69%)
Walk‐in	77 (31%)	76 (26%)
Recumbent patient transport	6 (2.4%)	11 (3.7%)
Other	3 (1.2%)	3 (1%)
Triage category		
1 (Immediate)	8 (3.5%)	17 (6.3%)
2 (Very urgent)	102 (45%)	124 (46%)
3 (Urgent)	109 (48%)	123 (46%)
4 (Non‐urgent)	8 (3.5%)	4 (1.5%)
Missing data	21 (9.2%)	26 (9.7%)

^a^ Median (Q1, Q3); *n* (%).

### 3.2 Main Results

Overall ED LOS decreased for patients living with frailty from a median of 352 min for the control group to 319 min for the post‐implementation group (*p* = 0.01). Time to physician assessment decreased in the post‐implementation group, from 44 to 31 min in the post‐implementation group (*p* < 0.001) (Table 2). The admission rate was unchanged at 59% in the control group and 60% in the post‐implementation group (*p* = 0.4) (Table 2). Both ED LOS and time to physician was reduced in triage acuity 1–3 subgroups, but increased in triage acuity 4 (less urgent) patients (Table 3).

**TABLE 2:** Emergency Department length of stay, admission, and waiting time to physician.

	Pre‐implementation group^a^	Post‐implementation group^a^	Difference (95% CI)	*p* ^b^
	Living with frailty (CFS > 4)	Living with frailty (CFS > 4)		
ED length of stay in minutes, median (IQR)	352 (266, 515)	319 (240, 458)	33 (33 to 43)	0.014
Waiting time to physician	44 (20, 94)	31 (15, 65)	13 (12 to 19)	< 0.001
Admission	145 (59%)	175 (60%)	1 (−7 to 9)	0.4

^a^ Median (Q1, Q3); *n* (%).

^b^ Wilcoxon rank sum test; Pearson's Chi‐squared test; Fisher's exact test.

**TABLE 3:** Emergency Department length of stay and waiting time to physician across different triage categories.

	Pre‐implementation group^a^	Post‐implementation group^a^	*p* ^b^
	Living with frailty (CFS > 4)	Living with frailty (CFS > 4)	
Triage category			
1 (Immediate)			
ED length of stay in minutes, median (IQR)	308 (235, 792)	260 (176, 362)	0.3
Wait time in minutes, median (IQR)	18 (3, 72)	14 (1, 44)	0.9
2 (Very urgent)			
ED length of stay in minutes, median (IQR)	350 (259, 487)	308 (240, 432)	0.09
Wait time in minutes, median (IQR)	30 (259, 487)	24 (13, 52)	0.2
3 (Urgent)			
ED length of stay in minutes, median (IQR)	378 (283, 530)	342 (238, 505)	0.1
Wait time in minutes, median (IQR)	74 (34, 122)	49 (21, 99)	0.006
4 (Less urgent)			
ED length of stay in minutes, median (IQR)	285 (179, 584)	402 (318, 1090)	0.4
Wait time in minutes, median (IQR)	69 (21, 92)	71 (16, 129)	0.5

^a^ Median (Q1, Q3).

^b^ Wilcoxon rank sum test; Pearson's Chi‐squared test; Fisher's exact test.

### 3.3 Displacement Effects

There were a total of 675 robust (CFS < 5) patients in the study with 323 pre‐implementation and 352 post‐implementation respectively. The mean age was 79 years of age and did not differ between the groups. There was a decrease in ED LOS from 317 to 303 min (*p* = 0.12) and a significant decrease in time to physician, from 54 min in the pre‐implementation group to 46 min for the post‐implementation group (*p* = 0.02). The admission rate was unchanged for robust patients at 41%.

## 4 Discussion

In this observational before and after study in a single‐center general ED, a clinical routine prioritizing patients based on CFS reduced ED LOS by 33 min and time to physicians by 13 min in frail patients. For patients in triage category 3 (urgent), the group with the largest proportion of patients living with frailty, the ED LOS was reduced by 25 min.

Reducing emergency department length of stay is particularly important for older patients, as prolonged stays increase mortality risk and hospital length of stay, creating further ED boarding [18, 20]. Decreases in ED LOS have been demonstrated in specialized geriatric EDs compared to ordinary EDs [31] and following interventions connected to geriatric teams where ED LOS significantly decreased from 19.1 to 12.7 h [32]. The first older people's Emergency Department in England demonstrated significantly lower ED LOS and waiting time to physician of 63 and 75 min respectively, compared to a main ED [33]. However, not all EDs have the resources to organize dedicated spaces and teams for geriatric patients, as is the case for our ED in Linköping, Sweden. These findings are therefore particularly relevant for general EDs operating without specialized geriatric resources, demonstrating that targeted frailty interventions can achieve meaningful improvements even in standard care settings.

The American College of Emergency Physicians' geriatric emergency department guidelines provide recommendations across six domains: staffing, equipment, education, policies and procedures, follow‐up care, and performance improvement measures [34]. However, the feasibility of the recommendations has been questioned considering most EDs worldwide lack the resources to provide the level of service described [35]. In the present study, we could observe reduced ED LOS by prioritizing patients living with frailty without the addition of specialized geriatric resources. Even if the existing evidence to support ED interventions to reduce adverse events for older ED patients is limited [26, 27], interventions during hospital stay have shown promise in improving functional status, decreasing in‐hospital length of stay, and readmissions [36]. Hence, reducing ED LOS may reduce morbidity by limiting the time spent in the ED and facilitating transfer to the hospital where the conditions to improve outcomes may be more favorable [20].

The prioritization of specific patient groups inherently raises concerns regarding potential adverse effects on care delivery to other patient groups. Our findings, however, indicate the opposite of a displacement effect with waiting time for physician being reduced post‐implementation and a trend toward shorter ED LOS for robust older patients as well. This may be caused by a phenomenon known as within‐unit spillover. Within‐unit spillover occurs when healthcare interventions targeting specific patients modify provider behavior throughout the unit, thereby affecting outcomes for non‐targeted patients receiving care from the same providers [37]. In this instance, knowledge about the new routine could have influenced staff members and ED work up was prioritized in older patients in general, even when they were not assessed as living with frailty. Thus, our study highlights that frailty alerts may improve lead times for vulnerable patients, without causing a negative displacement effect on robust older patients.

Our results suggest that reductions in LOS can be achieved through simple measures in a general ED, making way for implementing similar routines in other EDs. This simple intervention, along with targeted actions to decrease the risk of short‐term complications, could improve emergency care of older patients and additionally facilitate the overall ED patient flow.

In line with improving care for older ED patients, the patient aspect is vital to focus and build on. When Hörlin et al. [38] explored patient attitudes of being screened for frailty with CFS in our ED in Linköping, the results showed overall positive or neutral experiences. The purpose of the frailty assessment was however not perceived as entirely clear, highlighting the need for future research to investigate if a low‐resource frailty attuned routine has an impact on patient experience and reported outcomes, as well.

### 4.1 Limitations

We did not collect data on the remaining patients in the ED, nor some confounding factors affecting the LOS, such as the number of consult requests or radiology requests during the stay. To minimize the effect of external factors such as staffing, the data collection periods for the control‐ and post‐implementation groups were deliberately set in time periods with “ordinary” workflow, that is, not during holidays or extended vacations.

We did not find any displacement effects on robust patients, partly targeted by the intervention, which are also vulnerable to long ED LOS [19, 20] However, we cannot fully exclude displacement effects in other, less vulnerable patient groups. It was deemed unfeasible to collect data on other groups as the Swedish Review Authority requested patient consent for displacement analysis as well.

There is a risk of selection bias as more than a third of the intended inclusion opted out (*n* = 188, 39%). To counteract consistent exclusion of older patients in research, specifically involving routine data, the European Taskforce of Emergency Medicine advises ethics committees to carefully weigh the requirement for informed consent against the need for developed guidelines and improved care for older patients living with frailty [39].

There was a nonsignificant increase in ED LOS and waiting times for older patients living with frailty in triage category 4 (less urgent), but this group was small (4 patients pre‐implementation and 8 patients post‐implementation), which limits any conclusions about this group.

We did not measure the impact of the clinical routine on individual clinicians and hence we do not know if there were other confounding factors influencing prioritization between the pre‐implementation and post‐implementation period. This was a pragmatic study and the recommendation to prioritize older patients living with frailty was inherently vague to clinicians as there may be many aspects to consider when prioritizing ED patients.

## 5 Conclusion

Our study showed that frailty alerts, in a general ED without frailty units and geriatric teams, led to a significant decrease in ED LOS and waiting time to physician, without causing displacement effects on other older ED patients who did not have a frailty alert. Electronic frailty alerts as a clinical routine may improve ED flow whilst reducing adverse outcomes for vulnerable older patients.

## Author Contributions

S.M.E. and D.W. conceived and designed the study. D.W. and S.M.E. obtained permits. S.M.E. and J.W. conducted the data collection. S.M.E. analyzed the data and drafted the manuscript, J.W. and D.W. contributed to its revision.

## Funding

This research was funded by Region Ostergotland, a tax‐funded, public healthcare organization.

## Ethics Statement

The study was approved by the Swedish Ethical Review Authority (permit no. 2024‐05740‐01).

## Conflicts of Interest

The authors declare no conflicts of interest.

## Data Availability

Data are available upon reasonable request. There is no plan to share individual participant data. Personal data related to this study are available upon request. Electronic data are stored in a protected network storage space. The signed consent forms are stored in a locked space without access for unauthorized personnel.

## References

[1] M. Ukkonen , E. Jämsen , R. Zeitlin , and S. L. Pauniaho , “Emergency Department Visits in Older Patients: A Population‐Based Survey,” BMC Emergency Medicine 19, no. 1 (2019): 20. 30813898 10.1186/s12873-019-0236-3 PMC6391758

[2] G. George , “Effect of Population Ageing on Emergency Department Speed and Efficiency: A Historical Perspective From a District General Hospital in the UK,” Emergency Medicine Journal 23, no. 5 (2006): 379–383. 16627841 10.1136/emj.2005.029793 PMC2564089

[3] E. Burkett , “Emergency Medicine and Population Ageing: A Call to Action,” Emergency Medicine Australasia 36, no. 1 (2024): 4–5. 38228363 10.1111/1742-6723.14347

[4] Swedish Council on Health Technology Assessment , “Omhändertagande av äldre som inkommer akut till sjukhus—med fokus på sköra äldre” [Internet], accessed July 1, 2025, https://www.sbu.se/contentassets/5f0e7213e73b4369acd4874fd3dcbf89/akutvard_aldre.pdf.

[5] D. McIntyre and C. K. Chow , “Waiting Time as an Indicator for Health Services Under Strain: A Narrative Review,” Inquiry: The Journal of Health Care Organization, Provision, and Financing 57 (2020): 46958020910305. 10.1177/0046958020910305 PMC7235968 32349581

[6] G. Malmer , A. Fällman , R. Åhlberg , P. Svensson , E. Westerlund , and B. A. Ugglas , “Individual Patient Sociodemographic Characteristics Are Associated With Waiting Time to Physician Assessment in Seven Swedish Emergency Departments: An Observational 5‐Year Cohort Study of Emergency Department Visits in the Stockholm Region,” JACEP Open 6, no. 6 (2025): 100250. 41081285 10.1016/j.acepjo.2025.100250 PMC12514495

[7] M. R. Hofman , F. van den Hanenberg , I. N. Sierevelt , and C. R. Tulner , “Elderly Patients With an Atypical Presentation of Illness in the Emergency Department,” Netherlands Journal of Medicine 75, no. 6 (2017): 241–246. 28741583

[8] K. Erwander , K. Ivarsson , M. L. Olsson , and B. Agvall , “Elderly Patients With Non‐Specific Complaints at the Emergency Department Have a High Risk for Admission and 30‐Days Mortality,” BMC Geriatrics 24, no. 1 (2024): 5. 38172691 10.1186/s12877-023-04621-7 PMC10762826

[9] E. Burkett , M. G. Martin‐Khan , and L. C. Gray , “Comparative Emergency Department Resource Utilisation Across Age Groups,” Australian Health Review 43, no. 2 (2019): 194–199. 29224590 10.1071/AH17113

[10] G. Ogliari , F. Coffey , L. Keillor , et al., “Emergency Department Use and Length of Stay by Younger and Older Adults: Nottingham Cohort Study in the Emergency Department (NOCED),” Aging Clinical and Experimental Research 34, no. 11 (2022): 2873–2885. 36074240 10.1007/s40520-022-02226-5 PMC9453701

[11] C. L. Shenvi and T. F. Platts‐Mills , “Managing the Elderly Emergency Department Patient,” Annals of Emergency Medicine 73, no. 3 (2019): 302–307. 30287120 10.1016/j.annemergmed.2018.08.426

[12] N. R. Simon , A. S. Jauslin , R. Bingisser , and C. H. Nickel , “Emergency Presentations of Older Patients Living With Frailty: Presenting Symptoms Compared With Non‐Frail Patients,” American Journal of Emergency Medicine 59 (2022): 111–117. 35834872 10.1016/j.ajem.2022.06.046

[13] A. Clegg , J. Young , S. Iliffe , M. O. Rikkert , and K. Rockwood , “Frailty in Elderly People,” Lancet (London, England) 381, no. 9868 (2013): 752–762. 23395245 10.1016/S0140-6736(12)62167-9 PMC4098658

[14] S. Munir Ehrlington , E. Hörlin , R. Toll John , J. Wretborn , and D. Wilhelms , “Frailty Is Associated With 30‐Day Mortality: A Multicentre Study of Swedish Emergency Departments,” Emergency Medicine Journal 41, no. 9 (2024): 514–519. 39053972 10.1136/emermed-2023-213444 PMC11347252

[15] G. A. Solakoglu , “Evaluation of Factors Affecting the Length of Stay of Geriatric Patients in the Emergency Department” [Internet] (North Clinics of Istanbul, 2023), https://jag.journalagent.com/nci/pdfs/NCI‐59319‐RESEARCH_ARTICLE‐SOLAKOGLU.pdf. 10.14744/nci.2023.59319 PMC10500236 37719248

[16] P. Iozzo , N. Spina , G. Cannizzaro , et al., “Association Between Boarding of Frail Individuals in the Emergency Department and Mortality: A Systematic Review,” Journal of Clinical Medicine 13, no. 5 (2024): 1269. 38592117 10.3390/jcm13051269 PMC10932317

[17] H. L. Ellis , L. Dunnell , R. Eyres , et al., “What Can We Learn From 68 000 Clinical Frailty Scale Scores? Evaluating the Utility of Frailty Assessment in Emergency Departments,” Age and Ageing 54, no. 4 (2025): afaf093. 40253684 10.1093/ageing/afaf093 PMC12009543

[18] J. W. Joseph , N. Elhadad , M. L. P. Mattison , et al., “Boarding Duration in the Emergency Department and Inpatient Delirium and Severe Agitation,” JAMA Network Open 7, no. 6 (2024): e2416343. 38861262 10.1001/jamanetworkopen.2024.16343 PMC11167494

[19] L. Burgess , G. Ray‐Barruel , and K. Kynoch , “Association Between Emergency Department Length of Stay and Patient Outcomes: A Systematic Review,” Research in Nursing & Health 45, no. 1 (2022): 59–93. 34932834 10.1002/nur.22201

[20] M. Roussel , D. Teissandier , Y. Yordanov , et al., “Overnight Stay in the Emergency Department and Mortality in Older Patients,” JAMA Internal Medicine 183, no. 12 (2023): 1378–1385. 37930696 10.1001/jamainternmed.2023.5961 PMC10628833

[21] A. Alshibani , M. Alharbi , and S. Conroy , “Under‐Triage of Older Trauma Patients in Prehospital Care: A Systematic Review,” European Geriatric Medicine 12, no. 5 (2021): 903–919. 34110604 10.1007/s41999-021-00512-5 PMC8463357

[22] C. Poncet , P. N. Carron , V. Darioli , T. Zingg , and F. X. Ageron , “Prehospital Undertriage of Older Injured Patients in Western Switzerland: An Observational Cross‐Sectional Study,” Scandinavian Journal of Trauma, Resuscitation and Emergency Medicine 32, no. 1 (2024): 100. 39380009 10.1186/s13049-024-01271-5 PMC11462677

[23] K. Jang and Y. H. Seo , “Characteristics of Undertriaged Older Patients in the Emergency Department: Retrospective Study,” International Emergency Nursing 75 (2024): 101477. 38941741 10.1016/j.ienj.2024.101477

[24] E. Chong , B. Zhu , H. Tan , et al., “Emergency Department Interventions for Frailty (EDIFY): Front‐Door Geriatric Care Can Reduce Acute Admissions,” Journal of the American Medical Directors Association 22, no. 4 (2021): 923–928.e5. 33675695 10.1016/j.jamda.2021.01.083

[25] E. Chong , B. Zhu , S. H. X. Ng , et al., “Emergency Department Interventions for Frailty (EDIFY): Improving Functional Outcomes in Older Persons at the Emergency Department Through a Multicomponent Frailty Intervention,” Age and Ageing 51, no. 2 (2022): afab251. 35134848 10.1093/ageing/afab251

[26] A. Memedovich , B. Asante , M. Khan , et al., “Strategies for Improving ED‐Related Outcomes of Older Adults Who Seek Care in Emergency Departments: A Systematic Review,” International Journal of Emergency Medicine 17, no. 1 (2024): 16. 38302890 10.1186/s12245-024-00584-7 PMC10835906

[27] M. Conneely , S. Leahy , L. Dore , et al., “The Effectiveness of Interventions to Reduce Adverse Outcomes Among Older Adults Following Emergency Department Discharge: Umbrella Review,” BMC Geriatrics 22, no. 1 (2022): 462. 35643453 10.1186/s12877-022-03007-5 PMC9145107

[28] E. Moloney , M. R. O'Donovan , C. R. Carpenter , et al., “Core Requirements of Frailty Screening in the Emergency Department: An International Delphi Consensus Study,” Age and Ageing 53, no. 2 (2024): afae013. 38369629 10.1093/ageing/afae013 PMC10874925

[29] L. Brennan , K. Bentley , L. Edge , et al., “Flagging Frailty in the Emergency Department: A System to Identify and Visualise Frailty,” Age and Ageing 53, no. S4 (2024): afae178.151.

[30] C. McInnes , N. Moultrie , A. Wells , F. Campbell , E. Macdonald , and E. Tan , “1613 Improving Electronic Frailty Alerting in University Hospital Monklands: A Whole System Approach,” Age and Ageing 52, no. S2 (2023): afad104.027.

[31] C. J. Gettel , U. Hwang , A. T. Janke , et al., “An Outcome Comparison Between Geriatric and Nongeriatric Emergency Departments,” Annals of Emergency Medicine 82, no. 6 (2023): 681–689. 37389490 10.1016/j.annemergmed.2023.05.013 PMC10756927

[32] P. Heeren , E. Devriendt , S. Fieuws , et al., “Unplanned Readmission Prevention by a Geriatric Emergency Network for Transitional Care (URGENT): A Prospective Before‐After Study,” BMC Geriatrics 19, no. 1 (2019): 215. 31390994 10.1186/s12877-019-1233-9 PMC6686568

[33] C. Meechan , N. Navaneetharaja , S. Bailey , et al., “Evaluation of the First Older People's Emergency Department in England—A Retrospective Cohort Study,” Journal of Emergency Medicine 65, no. 1 (2023): e50–e59. 37355421 10.1016/j.jemermed.2023.04.003

[34] ACEP Geriatric , “Geriatric Emergency Department Guidelines” [Internet] (American College of Emergency Physicians, 2013), https://www.acep.org/siteassets/sites/geda/media/documnets/geda‐guidelines.pdf.

[35] R. D. Shih , C. R. Carpenter , V. Tolia , E. F. Binder , and J. G. Ouslander , “Balancing Vision With Pragmatism: The Geriatric Emergency Department Guidelines‐Realistic Expectations From Emergency Medicine and Geriatric Medicine,” Journal of Emergency Medicine 62, no. 5 (2022): 585–589. 35181186 10.1016/j.jemermed.2021.12.017

[36] Y. C. Wang , C. K. Liang , M. H. Chou , et al., “The Effectiveness of Frailty Intervention for Older Patients With Frailty During Hospitalization,” Journal of Nutrition, Health & Aging 27, no. 6 (2023): 413–420.10.1007/s12603-023-1924-y37357324

[37] I. Francetic , R. Meacock , J. Elliott , et al., “Framework for Identification and Measurement of Spillover Effects in Policy Implementation: Intended Non‐Intended Targeted Non‐Targeted Spillovers (INTENTS),” Implementation Science Communications 3, no. 1 (2022): 30. 35287757 10.1186/s43058-022-00280-8 PMC8919154

[38] E. Hörlin , D. Ekermo , D. Wilhelms , and A. C. Eldh , “Being Screened for Frailty in the Emergency Department: The Voice of Patients in an Exploratory Qualitative Study,” BMC Geriatrics 26, no. 144 (2026), 10.1186/s12877-026-06990-1. PMC12862903 41540358

[39] European Taskforce of Geriatric Emergency Medicine , “Promoting Uniform Data Reporting in Research on Older Individuals Living With Frailty in the Emergency Department: The Utstein Approach,” European Journal of Emergency Medicine, ahead of print, September 16, (2025), 10.1097/MEJ.0000000000001275. 40956183

